# Multifunctional hybrid nanoplatform based on Fe_3_O_4_@Ag NPs for nitric oxide delivery: development, characterization, therapeutic efficacy, and hemocompatibility

**DOI:** 10.1007/s10856-021-06494-x

**Published:** 2021-03-06

**Authors:** Joana Claudio Pieretti, Marcelly Chue Gonçalves, Gerson Nakazato, Ana Carolina Santos de Souza, Ariane Boudier, Amedea Barozzi Seabra

**Affiliations:** 1 Center for Natural and Human Sciences (CCNH), Federal University of ABC (UFABC), Santo André, SP Brazil; 2 Department of Microbiology, Universidade Estadual de Londrina, Londrina, PR Brazil; 3 Université de Lorraine, CITHEFOR, F-54000 Nancy, France

## Abstract

The combination of Fe_3_O_4_@Ag superparamagnetic hybrid nanoparticles and nitric oxide (NO) represents an innovative strategy for a localized NO delivery with a simultaneous antibacterial and antitumoral actions. Here, we report the design of Fe_3_O_4_@Ag hybrid nanoparticles, coated with a modified and nitrosated chitosan polymer, able to release NO in a biological medium. After their synthesis, physicochemical characterization confirmed the obtention of small NO-functionalized superparamagnetic Fe_3_O_4_@Ag NPs. Antibacterial assays demonstrated enhanced effects compared to control. Bacteriostatic effect against Gram-positive strains and bactericidal effect against *E. coli* were demonstrated. Moreover, NO-functionalized Fe_3_O_4_@Ag NPs demonstrated improved ability to reduce cancer cells viability and less cytotoxicity against non-tumoral cells compared to Fe_3_O_4_@Ag NPs. These effects were associated to the ability of these NPs act simultaneous as cytotoxic (necrosis inductors) and cytostatic compounds inducing S-phase cell cycle arrest. NPs also demonstrated low hemolysis ratio (<10%) at ideal work range, evidencing their potential for biomedical applications.

**Figure:**
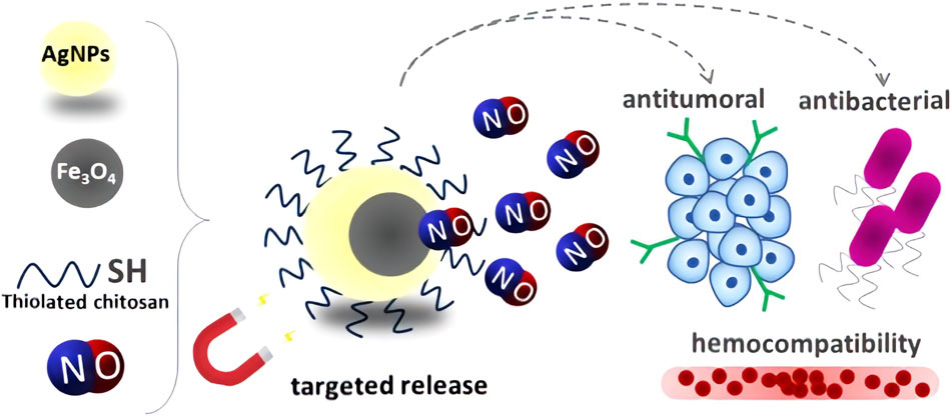
Targeted and hemocompatible nitric oxide-releasing multi-functional hybrid nanoparticles for antitumor and antimicrobial applications.

Targeted and hemocompatible nitric oxide-releasing multi-functional hybrid nanoparticles for antitumor and antimicrobial applications.

## Introduction

The use of nanoparticles (NPs) in biomedicine has been extensively reported in literature and continues to grow mostly due to their inherent physicochemical properties, the possibility of surface functionalization, in addition to antimicrobials and antitumoral properties [1]. In this regard, NP drug delivery systems lead to advantages over traditional drugs and their carrier systems, because the efficient delivery and release of the drug to the targeted location still requires improvement for the treatment of different diseases [2]. In the field of antibacterial treatments, the rapid emergence of antibiotic-resistance is a major challenge [3, 4]. In this sense, nanoparticulate-based treatment presents two outstanding points: (i) enhanced antibacterial activity compared to traditional therapies with antibiotics, and (ii) a decreased induction of resistance mechanisms [5]. Antitumoral treatments also present limitations. Chemotherapeutic drugs have to deal not only with induction of cell resistance, but also poor solubility in biological environment, low blood circulation time, and non-targeted distribution [6]. Thus, the research and development of efficient nanostructures are required for future advances in medical treatments [7].

Considering the promising applications of both antibacterial and antitumoral treatments, we developed a nitric oxide (NO) based delivery nanostructure comprising a magnetite (Fe_3_O_4_) and silver NPs (AgNPs) hybrid nanoplatform. Fe_3_O_4_ NPs have been studied for biomedical applications for, at least, the past three decades, mainly due to their intrinsic superparamagnetism at room and body temperatures, low toxicity, and good biocompatibility [8–11]. The biomedical applications of the Fe_3_O_4_ NPs include biosensors, bioseparation, hyperthermia therapy, and drug delivery [12–15]. Fe_3_O_4_ NPs have also been used in the development of multifunctional nanostructures, highlighting its combination with noble metal NPs [15]. Among the noble metal NPs, AgNPs present promising application in the field of biomedicine, standing out mostly for antibacterial applications in food storage, textile coating, and cosmetics [16–18]. More recently, AgNPs have been also shown as a promising system for antitumoral drug delivery, acting as an active platform, due to their intrinsic anticancer property [19].

Since NPs can also be functionalized with molecules of medical interest, in this work we proposed the synthesis of Fe_3_O_4_@Ag NPs that were further functionalized with thiol-modified chitosan (TCS), acting not only as a biocompatible layer, but also enabling the insertion of NO moieties and their spontaneous release [20]. NO is an endogenous signaling molecule, which plays pivotal roles in different physiological and pathological processes, such as vasodilation, neurotransmission, antibacterial activity, cell growth, and apoptosis [21, 22]. NO also presents antitumoral properties dependent on the applied concentration. At high concentrations, NO is able to kill tumor cells, but at low concentration, NO acts as a chemosensitizer, being promisor for the combination with active NPs and/or chemotherapeutics [22].

The synthesized NPs were characterized by several techniques, demonstrating the formation of the superparamagnetic hybrid NP, with 3% TCS coating, and a NO content of 1.21 ± 0.1 µmol per mg of Fe_3_O_4_@Ag/CS-NO NPs. The diffusion mechanism of NO from the NPs was evaluated and modeled by Korsmeyer–Peppas mathematical model. Additionally, was examined their antibacterial and antitumoral properties. Antibacterial activity was evaluated against two Gram-positive and one Gram-negative bacterial strain, and was demonstrated a synergistic effect between NPs and NO, resulting in a bacteriostatic effect against Gram-positive bacterial strains and a bactericidal effect against the Gram-negative one. Interestingly, pronounced effects were observed for tumoral cell lines, in which NO-releasing NPs demonstrated high efficacy. When compared to non-tumoral cell lines, cytotoxicity was evidenced between 5 and 40 µg mL^−1^ of Fe_3_O_4_@Ag/CS-NO NPs, and interestingly was more pronounced for tumoral cell lines. Flow cytometry revealed that Fe_3_O_4_@Ag/CS-NO NPs induced cell cycle arrest, indicated by the decrease of cells in the G0/G1 phase, and an increase in S phase. Apoptosis assays revealed that the necrosis pathway prevailed, especially at higher concentrations (>40 µg mL^−1^). As blood compatibility comprehends an important preclinical evaluation, the hemolytic toxicity of the NPs was studied and indicated that, at the concentration range pointed in cell viability assays, Fe_3_O_4_@Ag/CS-NO NPs presented minimal hemolysis of whole blood (5.4%), suggesting promising applications of the developed delivery system in the biomedical field. To the best of our knowledge, this is the first report to present the synthesis of NO-releasing NPs comprise of Fe_3_O_4_@AgNPs/CS-NO, and their antibacterial, antitumoral, and blood compatibility aspects.

## Materials and methods

### Chemicals

Resazurin sodium salt (alamar blue), thioglycolic acid, iron chloride II tetrahydrate (FeCl_2_ ∙ 4H_2_O), copper chloride II (CuCl_2_), N-(3-dimethylaminopropyl)-N′-ethylcarbodiimide hydrochloride, sodium nitrite (NaNO_2_), l-glutathione, and chitosan (CS) (75% deacetylation, medium molecular weight), propidium iodide (PI) and Annexin V (FITC) were acquired from Sigma—Aldrich, St. Louis, MO, USA. Iron chloride III hexahydrate (FeCl_3_ ∙ 6H_2_O), dimethyl sulfoxide, ammonium hydroxide (NH_4_OH), acetic acid (HAc), hydrochloric acid (HCl), silver nitrate (AgNO_3_), sodium hydroxide (NaOH) were obtained from Synth, Diadema, SP, Brazil. Powdered green tea (Camellia Sinensis) was obtained from Sumioka Shokuhin Kabushikikaisha, Hiraguti, Japan. All experiments were carried out using analytical grade water from Millipore Milli-Q Gradient filtration system (Millipore, 18.2 MΏ, USA).

### Synthesis of Fe_3_O_4_@Ag NPs, TCS, and Fe_3_O_4_@Ag NPs/TCS

Fe_3_O_4_@Ag NPs were prepared through chemical co-precipitation and green tea reduction of Ag^+^ ions, as previously reported [23]. TCS was prepared by the conjugation of thioglycolic acid in the presence of 1-etil-3-(3-dimethylaminopropyl)carbodiimide [20]. For the obtainment of Fe_3_O_4_@Ag NPs/TCS, Fe_3_O_4_@Ag NPs were dispersed in water (20% wt/wt) for 1 h in ultrasound bath, followed by the addition of TCS dissolved in acetic acid 1%. The final suspension was stirred for 2 h. Fe_3_O_4_@Ag NPs/TCS were magnetically separated, washed three times with water and freeze dried (Fig. S1).

### Characterization of Fe_3_O_4_@Ag/TCS NPs and control NPs

The structure of Fe_3_O_4_@Ag/TCS NPs was characterized by X-ray diffraction (XRD) and X-ray photoelectron spectroscopy (XPS). The morphology, size, and organic content of the synthesized NPs were characterized by scanning electron microscopy (SEM) coupled with energy dispersive X-ray fluorescence spectrometry, transmission electron microscopy (TEM), and atomic force microscopy (AFM). Hydrodynamic size, polydispersity index, and surface zeta potential of Fe_3_O_4_ NPs, Fe_3_O_4_@Ag NPs, and Fe_3_O_4_@Ag/TCS NPs were analyzed by dynamic light scattering (DLS). The organic content on the surface of Fe_3_O_4_@Ag NPs and Fe_3_O_4_@Ag/TCS NPs was quantified through thermogravimetric analyses. The magnetic properties of Fe_3_O_4_ NPs, Fe_3_O_4_@Ag NPs, and Fe_3_O_4_@Ag/TCS NPs were evaluated using a Quantum Design SQUID MPMS3 magnetometer. Detailed information of each employed technique is found in the Supplementary File.

### Formation of Fe_3_O_4_@Ag/CS-NO NPs

Free thiol (SH) groups on the surface of Fe_3_O_4_@Ag/TCS NPs were nitrosated by reacting the NPs with an aqueous solution of NaNO_2_ at pH 6, followed by further removal of NaNO_2_ excess with water. Total NO release from *Fe*_*3*_*O*_*4*_*@Ag/CS-NO NPs* was quantified through amperometric measurements with the Free Radical Analyzer TBR 1025 (*World Precision Instruments*, FL, USA), using a specific NO probe (ISO-NOP 2 mm). A detailed procedure is in the Supplementary File.

### In vitro diffusion of NO from Fe_3_O_4_@Ag/CS-NO NPs

To evaluate the in vitro diffusion of NO from Fe_3_O_4_@Ag/CS-NO NPs a vertical Franz diffusion cell (*Hanson Research Corporation*, CA, USA) was employed with a hydrophilic nitrocellulose membrane with 50 nm porosity separating the donor and receptor compartments (*Merck Millipore*, MA, USA) [24]. The receptor compartment of the cell was filled with 7 mL of PBS and kept in constant stirring at 37 °C. The donor compartment was filled with 10 mg of Fe_3_O_4_@Ag/CS-NO NPs dispersed in water. Aliquots of 500 µL were taken each 30 min from the receptor compartment and the same volume was reposed by the injection of 500 µL pf PBS. The amount of NO content was quantified with the Free Radical Analyzer TBR 1025 with a specific NO probe. Mathematical models were applied to modeling the NO release from Fe_3_O_4_@Ag/CS-NO NPs.

### Antibacterial assays

Gram-negative *Escherichia coli (E. coli)* ATCC 25922, and Gram-positive *Staphylococcus aureus* (*S. aureus*) ATCC 25923, and *Streptococcus mutans* (*S. mutans*) UA159 were employed for antimicrobial activity screening using CLSI 2018 (Clinical and Laboratory Standards Institute) standard. Minimum inhibitory concentration (MIC), minimum bactericidal concentration (MBC), and time-kill curves were performed for antimicrobial activity testing of Fe_3_O_4_@Ag, Fe_3_O_4_@Ag/TCS, and Fe_3_O_4_@Ag/CS-NO NPs. These experiments were based on triplicate assays and accomplished following CLSI guidelines [25]. Detailed information about the antibacterial experiments can be found in Supplementary file.

### Cells culture, treatment, and evaluation of viability

The human prostate carcinoma (PC3) cell line was provided by Prof. Marcelo Bispo de Jesus (State University of Campinas, Brazil). The human osteocarcinoma (MG63) and mouse preosteoblastic (MC3T3-E1) cell lines were provided by Prof. Juliana Marchi (Federal University of ABC, Brazil), respectively. The human foreskin fibroblast (HFF-1) cell line was provided by Dr. Giselle Zenker Justo (Federal University of São Paulo, Brazil). African Green monkey (Cercopithecus aethiops) kidney normal cells (Vero) were provided by Prof. Christiane Bertachini Lombello (Federal University of ABC, Brazil). The cells were cultured in RPMI 1640 medium (PC3), Alpha-MEM (MC3T3-E1), and Dulbecco’s DMEM with high (HFF-1, Vero) or low (MG63) glucose. All media were supplemented with 10% (v/v) FBS, 100 U/mL of penicillin, and 100 μg/mL of streptomycin. For HFF-1 cells, Dulbecco’s DMEM was supplemented with 15% (v/v) FBS and 1 mmol L^−1^ sodium pyruvate while MC3T3-E1 cells were growth in absence of ascorbic acid. Cultures were maintained at 37 °C in a humidified incubator with a 5% CO_2_ atmosphere. Cells were passaged every 2–3 days, and viability and cell density were periodically checked by the trypan blue dye exclusion test.

The in vitro effects of Fe_3_O_4_@Ag, Fe_3_O_4_@Ag/TCS, and Fe_3_O_4_@Ag/CS-NO NPs against viability of the cancer and non-tumor cells was verified through Alamar Blue assay [26]. Cells were seeded at a density of 8.0 × 10^3^ cells per well, in 96-well plate for 24 h, and treated with the following concentrations of each NP: 5, 10, 40, 80, 100, 150, and 200 µg mL^−1^. After 24 h incubation, 20 µL of Alamar Blue solution (0.3 mg mL^−1^) was added to each well and incubated for 3 h. The fluorescence was analyzed at 530/25 nm excitation length and 590/35 nm emission length using a plate reader (Synergy HT Multi-Mode Microplate Reader, Biotek, USA). Cells without treatment were used as a control for 100% cell viability.

### Analysis of cell cycle distribution and apoptotic incidence by flow cytometry

For cell cycle analysis, MG63 cells were with Fe_3_O_4_@Ag/CS-NO NPs at the concentrations of 5, 40, and 100 µg mL^−1^. Cells were seeded at a density of 1.4 × 10^6^ cells per 100 mm tissue culture dish and were treated similarly to the previous experiments. After 24 h treatment, cells were detached with trypsin, collected, washed in PBS, and subsequently fixed in paraformaldehyde (1% in PBS) and ethanol 70%. Following, cells were resuspended in PBS and incubated with PI staining solution (5 µL RNase A 10 mg/mL, 20 µL PI 1 mg/mL) in the darkness for 30 min. BD FACSCanto™ II Cell Analyzer (Becton Dickinson, San Diego, NJ, USA) and FACSDiva software were used for acquisition. For apoptosis assay, cells treated with Fe_3_O_4_@Ag/CS-NO NPs were detached with trypsin, collected, and centrifuged. Cells were resuspended in 100 µL of Annexin V-binding buffer and stained with 3 µL of Annexin V-FITC and 1 µL of PI. Flow cytometry analyses were performed similarly to the cell cycle analyses. Cisplatin (50 µmol L^−1^) was used as positive control, and pure culture medium as negative control. All data were analyzed using Flowjo 10.6.1 (FlowJo, LLC, USA).

### Hemolysis of whole blood

The experiments were performed in accordance with the European Community guidelines (2010/63/EU) for the use of experimental animals. Arterial blood samples were collected from rats (Male Wistar), which were anesthetized with isoflurane (4% in oxygen 2 L min^−1^) 15 min before sampling. The blood was collected from abdominal artery, in Vacutainer tubes with heparin lithium using a short catheter. Before use, whole blood was diluted in the proportion of 1 mL of blood to 1.25 mL of NaCl 0.9% solution. After, 1 mL of diluted blood solution was added to 24 mL of NaCl 0.9% solution, in order to obtain work blood solution.

Fe_3_O_4_ NPs, Fe_3_O_4_@Ag NPs, Fe_3_O_4_@Ag/TCS NPs, and Fe_3_O_4_@Ag/CS-NO NPs were dispersed in concentrations from 60 to 1200 µg mL^−1^ in NaCl 0.9% medium, for 1 h in ultrasound bath. Then, NPs were diluted into work blood solution, in the proportion of 500 µL of blood solution and 100 µL of NPs. The solutions (blood + NPs) were incubated for 2 h, at 37 °C in a water bath WNB/WNE/WPE (Memmert, Schwabach, Germany). The resulting suspension was centrifuged at 4000 rpm for 10 min using a microtube centrifuge MiniSpin Plus (Eppendorf) and the hemolysis ratio (HR) was evaluated by spectrophotometric measurements against negative and positive controls (NaCl 0.9% and water, respectively), using a spectrophotometer UV-1800 (Shimadzu, France) at 541 nm in plastic cuvettes.

## Results and discussion

### Characterization of Fe_3_O_4_@Ag/TCS NPs and control NPs

**Fig. 1:**
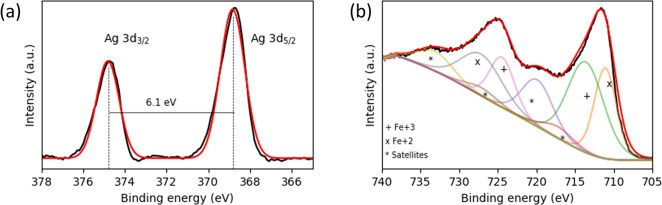
**a** XPS spectrum of Fe 2p region of Fe_3_O_4_ NPs and **b** Ag 3d region of Fe_3_O_4_ NPs

In this work, we synthesized Fe_3_O_4_ NPs by co-precipitation followed by the reduction of Ag^+^ by green tea extract on the surface of Fe_3_O_4_ NPs leading to the formation of Fe_3_O_4_@Ag NPs, as previous reported [23, 27]. The surface of Fe_3_O_4_@Ag NPs was coated with TCS, by thiolation of CS backbone [20], leading to the formation of Fe_3_O_4_@Ag/TCS NPs. The prepared NPs were characterized, as described herein. The structure of the uncoated Fe_3_O_4_@Ag NPs was previously confirmed, evidencing the formation of a structure composed of 87% of Fe_3_O_4_ NPs and 13% of AgNPs through XRD analyses, which was maintained in Fe_3_O_4_@Ag/TCS NPs [23]. The presence of AgNPs and Fe_3_O_4_ was also confirmed by XPS, as shown in Fig. 1a and 1b, respectively. The high-resolution Ag spectrum evidences an efficient green tea reduction of Ag^+^ and formation of metallic AgNPs, as Ag *3d*_*3/2*_ and Ag *3d*_*5/2*_ core level binding energies are located at 374.7 and 368.8 eV, respectively, presenting a split of 6.1 eV between the two peaks, characteristic of the referred Ag^0^ structure [28]. The fitted Fe 2p region shown in Fig. 1b indicates the presence of Fe^2+^ and Fe^3+^ states in a ratio of, approximately, 40:60% (^2+^/^3+^), and a 13.5 eV splitting between Fe *2p*_*3/2*_ and Fe *2p*_*1/2*_, evidencing a structure between magnetite and maghemite [29, 30].

**Fig. 2:**
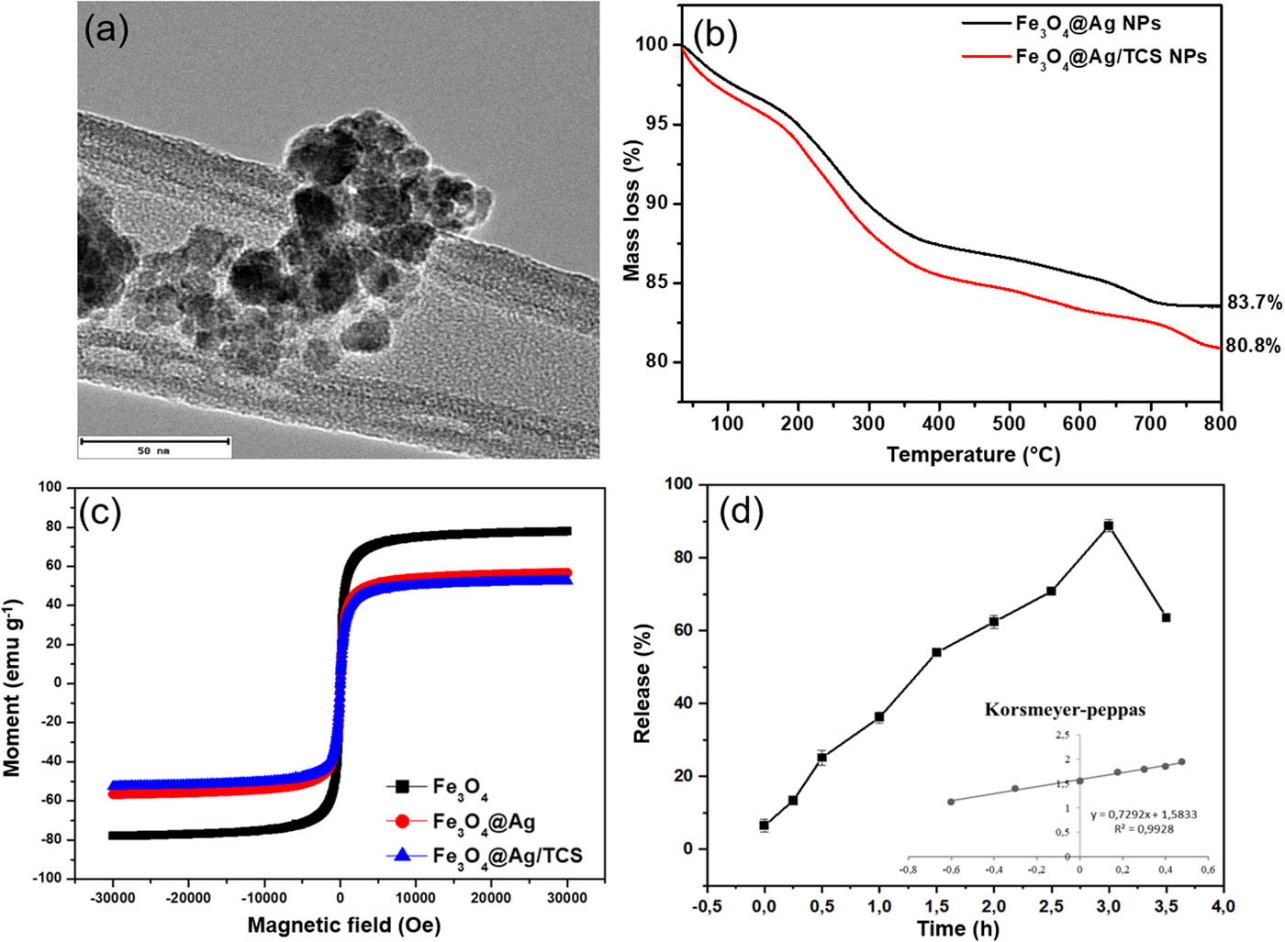
**a** Representative TEM image of Fe_3_O_4_@Ag/TCS NPs, scale bar indicates 50 nm; **b** thermogram of Fe_3_O_4_@Ag NPs and Fe_3_O_4_@Ag/CS-NO NPs; **c** hysteresis curves at room temperature for Fe_3_O_4_@Ag, Fe_3_O_4_@Ag/TCS, and Fe_3_O_4_@Ag/CS-NO; **d** Diffusion profiles of NO from Fe_3_O_4_@Ag/CS-NO NPs modeled by Korsmeyer–Peppas. The error bars represent the average between three independent experiments

The morphology of the Fe_3_O_4_@Ag/TCS NPs was investigated by TEM (Fig. 2a). The NPs presented a spherical shape with an average size of 17.3 ± 2.8 nm, at solid state. The results are in accordance to SEM and AFM analyses (Fig. S2), confirming a spherical morphology, the elementary composition, and the polymeric coating of the NPs. Similar NPs reported in literature presented spherical shape and diverse diameters depending on the synthesis route and on the coating thickness [31, 32]. The hydrodynamic size (% number), polydispersity index, and zeta potential of the Fe_3_O_4_ NPs, Fe_3_O_4_@Ag NPs, and Fe_3_O_4_@Ag/TCS NPs were evaluated and compared through DLS technique. Fe_3_O_4_ NPs presented high levels of aggregation and polydispersity (350.6 ± 40.3 nm, PDI 0.54 ± 0.03), as expected for uncoated NPs, which decreased after the Ag coating, leading to an average size of 25.97 ± 2.5 nm and PDI of 0.46 ± 0.03. After TCS coating, Fe_3_O_4_@Ag/TCS NPs hydrodynamic size increased to 81.8 ± 3.8 nm due to the polymeric layer, although the PDI decreased to 0.20 ± 0.01, indicating more homogeneous NP size distribution. The zeta potential for Fe_3_O_4_ NPs, Fe_3_O_4_@Ag NPs, and Fe_3_O_4_@Ag/TCS NPs were −8.56 ± 1.00, −32.73 ± 0.31, −19.40 ± 1.00, respectively.

The organic and polymeric contents were investigated by thermogravimetric analysis. The thermograms of Fe_3_O_4_@Ag NPs and Fe_3_O_4_@Ag/TCS NPs are shown in Fig. 2b. An initial mass loss is observed at *ca*. 100 °C referent to the water content, followed by the decomposition of the organic content from green tea molecules, such as catechin and other polyphenols, adsorbed on the surface of the hybrid NP, and the decomposition of both thiolated chitosan and green tea extract molecules on the Fe_3_O_4_@Ag/TCS NPs. From the mass loss difference, it was possible to verify the presence of 28% derived from phytochemicals of the green tea extract and 3% of TCS attached to the surface of the NPs [33].

The influence of the diamagnetic layers on the superparamagnetic behavior of the NPs was evaluated with a magnetometer at 300 K, and the resultant hysteresis loops for Fe_3_O_4_ NPs, Fe_3_O_4_@Ag NPs, and Fe_3_O_4_@Ag/TCS NPs are shown in Fig. 2c. A superparamagnetic behavior was observed for all NPs, evidenced by the zero coercivity (Hc = 0) and zero magnetic remanence (Mc = 0) [34]. The magnetization saturation (Ms) values obtained were 76.8, 56.1, and 56.9 emu g^−1^ for Fe_3_O_4_ NPs, Fe_3_O_4_@Ag NPs, and Fe_3_O_4_@Ag/TCS NPs, respectively. The Ms values obtained for Fe_3_O_4_ NPs are similar to the reported for this material in the literature, with values from 62 to 90 emu g^−1^, confirming the magnetic properties expected for this nanomaterial [27, 35]. For Fe_3_O_4_@Ag NPs and Fe_3_O_4_@Ag/TCS NPs, lower Ms values are observed, in accordance to the addition of diamagnetic layers (Ag, phytochemicals derived from green tea, and TCS) [36]. A slight difference in the Ms values is observed when comparing Fe_3_O_4_@Ag NPs and Fe_3_O_4_@Ag/TCS NPs, which is in accordance to thermogravimetric analysis, as a small percentage of polymeric content (TCS) was added. Thus, all NPs kept their superparamagnetic behavior, being promissory for targeted biomedical treatments.

### Formation of Fe_3_O_4_@Ag/CS-NO NPs

Free thiol groups of TCS on the surface of Fe_3_O_4_@Ag/TCS NPs were nitrosated by reacting with NaNO_2_ in slight acid medium, in which nitrous acid is the nitrosating agent, yielding S-nitroso (S-NO) moieties in the TCS [24]. This process led to the formation of S-nitrosothiol groups covalently linked to the CS backbone, in the coating of the NPs (*Fe*_*3*_*O*_*4*_*@Ag/CS-NO NPs*). The obtained nitrosated NPs act as spontaneous NO donor, as described below.

### In vitro diffusion of NO from Fe_3_O_4_@Ag/CS-NO NPs

Figure 2d shows the NO release profile from the nitrosated NPs (Fe_3_O_4_@Ag/CS-NO NPs). It was possible to verify that NO was linearly released during 3 h of analysis, reaching 90% of the total amount of NO (1.21 μmol per mg of NP). It is important to highlight that NO content was measured through the NO release from S-NO bonds on the surface of the NPs. Thus, the concentrations of NO, S-NO, and SH are equimolar. At 3.5 h, NO release decreases, as evidenced in Fig. 2d. We have recently reported that the NO release profile from S-nitroso-chitosan (without the nanoplatform) was comprised of an initial rapid NO release for 1 h [37], whereas Fe_3_O_4_@Ag/CS-NO NPs presented an initial burst of NO release after 3 h of monitoring.

To confirm the diffusion mechanism, the curves were fitted using different mathematical models (order zero, first order, Higuchi, Hixson–Crowel, and Korsmeyer–Peppas) as previously reported in literature [38, 39]. The resulting correlation coefficient (*R*^2^) was 0.993 for Korsmeyer–Peppas indicating that NO release from Fe_3_O_4_@Ag/CS-NO NPs was better modulated by this model. The Korsmeyer–Peppas mathematical model indicates that NO was released by a non-Fickian diffusion process, or concentration independent, verified by the *n* = 0.73. Moreover, this model proposes that the NO was not only being diffused, but also describes a release based on the polymeric chain relaxation, which is in according to the designed polymeric coated NPs [38, 39].

### Antibacterial assay

Considering the known antibacterial properties of individual AgNPs, CS, and NO, was evaluated if their combination in a single nanomaterial might enhance the antibacterial effect, compared to the individual components. Thus, the antibacterial effect of Fe_3_O_4_@Ag NPs, Fe_3_O_4_@Ag/TCS, and Fe_3_O_4_@Ag/CS-NO was evaluated. Table 1 shows the MIC and MBC values obtained. The results indicated a synergism between Ag and NO in the same NP (Fe_3_O_4_@Ag/CS-NO), leading to a higher toxicity against both *S. mutants* and *S. aureus*, as lower values of MIC were observed when compared to the other NPs. Regarding the effects against *E. coli*, results were similar to the Fe_3_O_4_@Ag NPs.

**Table 1:** Minimum inhibitory concentrations (MIC) and minimum bactericidal concentration (MBC) values (µg mL^−1^ of NPs) for bacterial strains after 24 h of incubation with Fe_3_O_4_@Ag NPs, Fe_3_O_4_@Ag/TCS e Fe_3_O_4_@Ag/CS-NO

	*E. coli* 25922		*S. mutans* UA159		*S. aureus* 25923	
	MIC	MBC	MIC	MBC	MIC	MBC
Fe_3_O_4_@Ag NPs	125	125	500	>500	250	500
Fe_3_O_4_@Ag/TCS	250	250	500	>500	250	500
Fe_3_O_4_@Ag/CS-NO	125	125	250	>500	125	500

**Fig. 3:**
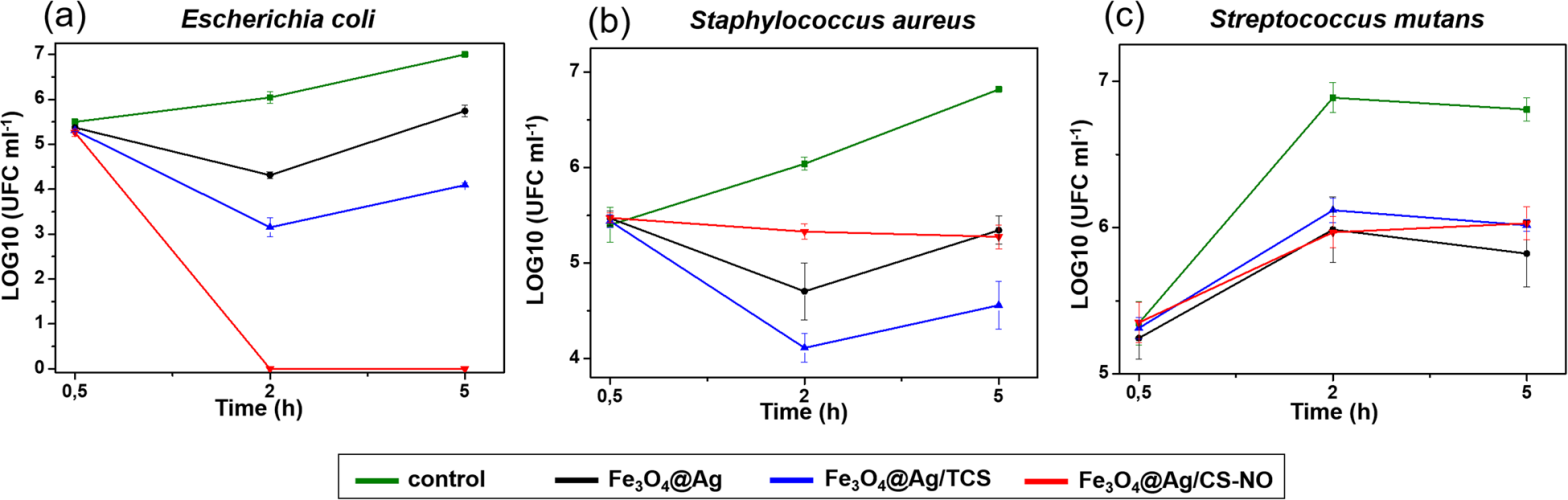
Time-kill curves for **a** *E. coli*, **b** *S. aureus*, and **c** *S. mutans*. The green lines represent the control, in black Fe_3_O_4_@Ag NPs, in blue Fe_3_O_4_@Ag/TCS NPs, and in red Fe_3_O_4_@Ag/TCS-NO NPs. The error bars represent the average between three independent experiments

In order to better understand antibacterial effect of each, time-kill curves were performed using MIC, as shown in Fig. 3. Time-kill curves evidences a bacteriostatic effect for all NPs during 5 h of treatment against both *S. mutants* and *S. aureus*, demonstrated through a reduced growth when compared to control strains. Regarding *E. coli*, a bacteriostatic effect was observed for Fe_3_O_4_@Ag NPs and Fe_3_O_4_@Ag/TCS. While a bactericidal effect was confirmed for the Fe_3_O_4_@Ag/CS-NO after 2 h of treatment [40]. These results indicate promising use of these NPs in the treatment of infectious diseases, especially because the magnetic nanomaterial can be guided to the targeted location in combination with antibiotics. Moreover, for *E. coli*, the Fe_3_O_4_@Ag/CS-NO might be administered in multiple doses of lower concentrations, minimizing the side effects [41]. Still, these NPs might find potential applications

### Cytotoxicity

**Fig. 4:**
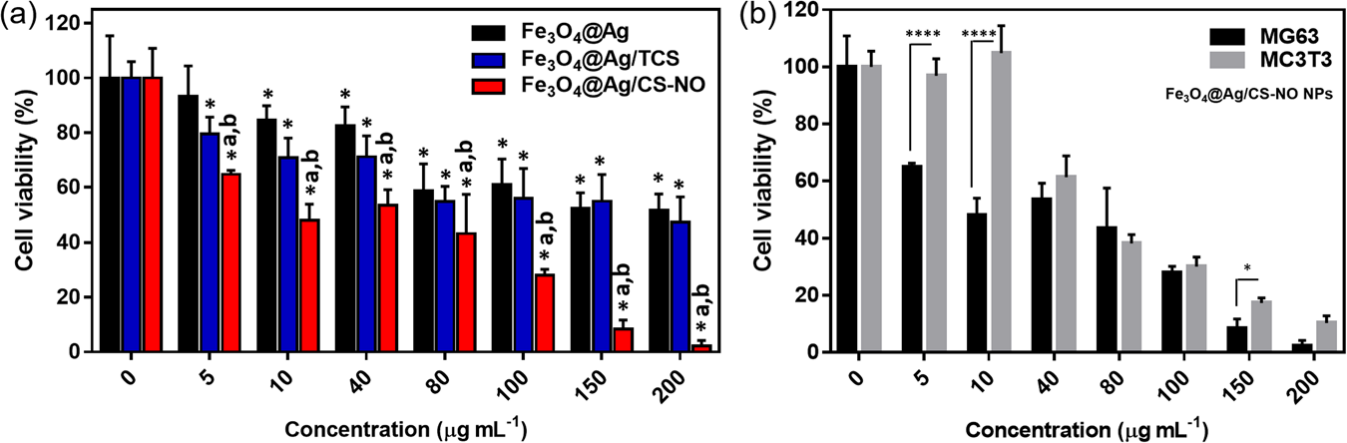
Effects of NPs on tumoral and non-tumoral cell lines. **a** Viability of MG63 cells treated with different concentrations of Fe_3_O_4_@Ag, Fe_3_O_4_@Ag/TCS, and Fe_3_O_4_@Ag/CS-NO for 24 h was assayed by alamar blue test. Asterisk indicates significative difference (*p* < 0.05) between treatments and control. a,b indicates a statistically significant difference (*p* < 0.05) between “a” Fe_3_O_4_@Ag and Fe_3_O_4_@Ag/CS-NO, and “b” Fe_3_O_4_@Ag/TCS and Fe_3_O_4_@Ag/CS-NO. **b** Cell viability of MG63 and MC3T3 cells treated with Fe_3_O_4_@Ag/CS-NO at different concentrations for 24 h assayed through alamar blue test. *P < 0.0274, and ****P < 0.0001, showed by Two-Way ANOVA

The cytotoxic effects of the NPs were evaluated against MG63 tumoral cell line and compared to MC3T3-E1 non-tumoral cells (Fig. 4). Moreover, the cytotoxicity was verified against PC3, HFF-1 and Vero cells lines, as shown in Fig. S3 and described in Supplementary File. Figure 4a shows the comparison between Fe_3_O_4_@Ag NPs, Fe_3_O_4_@Ag/TCS NPs e Fe_3_O_4_@Ag/CS-NO NPs against MG63, after 24 h treatment. A concentration-dependent cytotoxicity was observed for all tested NPs, although Fe_3_O_4_@Ag NPs and Fe_3_O_4_@Ag/TCS did not present high toxicity even at the highest evaluated concentration (200 µg mL^−1^), presenting a cell viability of 60% approximately, at this concentration. Antitumoral activity has been reported for NO [42], and might act as sensitizing agent. In previous reported we have demonstrated that S-nitrosoglutathione combined with AgNPs had a synergistic toxicity effect to cancerous cells [43]. Therefore, in this work, was hypothesized that the combination of AgNPs and NO in a single nanostructure would have potent and synergist cytotoxic effects. As expected, Fig. 4 shows that Fe_3_O_4_@Ag/CS-NO NPs demonstrated higher toxicity, compared to Fe_3_O_4_@Ag NPs and Fe_3_O_4_@Ag/TCS NPs, evidencing an effective combination of NO and NPs [42, 44]. A significant statistical difference (*p* < 0.05) of cells treated with Fe_3_O_4_@Ag/CS-NO NPs was verified for all evaluated concentrations, and the viability of MG63 cells was almost 0% at 200 µg mL^−1^.

The cytotoxicity of Fe_3_O_4_@Ag/CS-NO NPs for MG63 and MC3T3 cell lines were compared. As shown in Fig. 4b, compared to the tumoral cell line, the cytotoxicity was significant lower (*p* < 0.05) for MC3T3 cells at 5 and 10 µg mL^−1^, and slightly lower at 40 µg mL^−1^. For the highest concentrations (80–200 µg mL^−1^) were too cytotoxic for both cell lines. In this sense, a promising working range is shown between 5 and 40 µg mL^−1^, in which a higher cytotoxicity was more evident only for the tumoral cell line. Additionally, the superparamagnetic properties of Fe_3_O_4_@Ag/CS-NO NPs allow these to be targeted directly to the tumor site, decreasing possible side effects [45].

### Flow cytometry

**Fig. 5:**
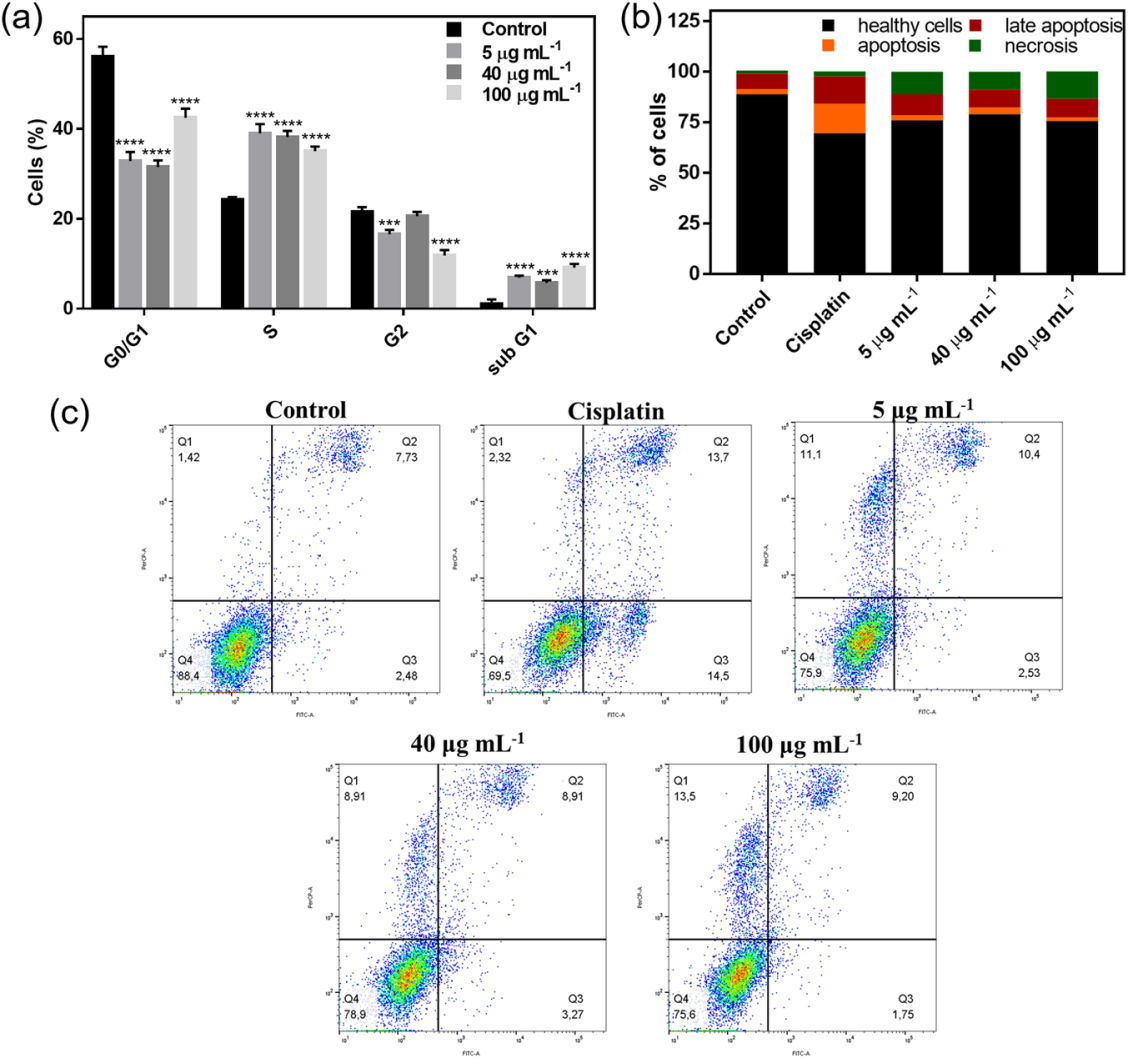
Effects of NPs in cell cycle and apoptosis induction in MG63. **a** Cell cycle analysis indicating cytostatic effect of Fe_3_O_4_@Ag/CS-NO NPs at different concentrations (***P < 0.009, and ****P < 0.0001, in relation to control, evaluated by Two-Way ANOVA); cell apoptosis assay (PI/Annexin V) by flow cytometry indicated by (**b**) percent of cells (healthy, undergoing apoptosis, late apoptosis and necrosis); **c** scatterplots of each treatment group of MG63 cells

Considering the promising results for Fe_3_O_4_@Ag/CS-NO NPs in cytotoxicity assays, and in order to better understand of its effects on MG63 tumoral cell line, flow cytometry was used in cell cycle and apoptosis assays to evaluate the influence of the treatment in the induction of cell arrest and/or cell death by apoptosis. Figure 5a shows the percentage of MG63 cells at G0/G1, S, G2, and sub-G1 phases, after 24 h treatment at different concentrations of Fe_3_O_4_@Ag/CS-NO NPs. The results showed that all concentrations the Fe_3_O_4_@Ag/CS-NO NPs induced a reduction in the percentage of cells at G0/G1 and G2/M phases followed by a simultaneous increase in S-phase population. Thus, the results demonstrated that Fe_3_O_4_@Ag/CS-NO NPs presents a cytostatic effect inducing cell cycle arrest at S phase for MG63 cells. During cell cycle, S phase checkpoint protects the replication of damaged DNA inducing the cell cycle arrest and inhibiting the ongoing the DNA synthesis [46, 47]. DNA damage might be induced, for example, by external genotoxic effects, suggesting potential genotoxicity of Fe_3_O_4_@Ag/CS-NO NPs against MG63 cells [48]. This result is expected considering the combined treatment of AgNPs and NO. Previous works evidenced that NO is able to induce cell cycle arrest in other cell lineages, while AgNPs are well known to induce oxidative stress, DNA damage and have also been reported to arrest cell cycle at S phase [48–51].

Since sub-G1 population have been shown significantly increased after Fe_3_O_4_@Ag/CS-NO NPs treatment, indicating the presence of dead cells, it was analyzed the possible induction of apoptotic cell death through flow cytometry. Figure 5b shows the percentage of cells in early apoptosis (AN^+^/PI^−^), late apoptosis (AN^+^/PI^+^) and necrosis (AN^−^/PI^+^), quantified using the scatterplots presented in Fig. 5c. Considering total apoptotic population (early + late apoptosis) it is possible observed that for Fe_3_O_4_@Ag/CS-NO NPs treatment at 5 and 40 μg mL^−1^ we found only a 3% increase in apoptotic cells in relation to control while in the higher concentration, no increase in the number of apoptotic cells was detected. However, in all treatments Fe_3_O_4_@Ag/CS-NO NPs induced at least 10 times increase in the number of necrotic cells compared to control reaching the highest increase for 100 μg mL^−1^. AgNPs have already been reported to induce both apoptosis and necrosis in human breast adenocarcinoma (MCF-7) and in L929 fibroblast cells depending on the size and concentration [52–54]. Moreover, NO is well described in literature as a tumor sensitizer, which might have potentialized the effects when treating MG63 with the combined NP [55].

Interestingly, besides the increase in necrotic population it is important to highlight that even at higher concentration of Fe_3_O_4_@Ag/CS-NO NPs the percentage of necrotic cells was low (13.5%) considering that this treatment induced reduction in viable cells of around 70%. Thus, in this condition a significantly percentage in cell viability reduction seems associated to the ability of NP to cause cell cycle arrest. The cytostatic effect is also important for the reduction in viability in the treatments with smaller concentrations of Fe_3_O_4_@Ag/CS-NO NPs [48–51].

For future research, the amounts of NO, AgNPs and Fe_3_O_4_ NPs contents in the final Fe_3_O_4_@Ag/CS-NO NPs can be tailored to treat different types of tumors, achieving the desired cell death pathway or decreasing cell proliferation. Moreover, Fe_3_O_4_ NPs are well-known for their magnetic properties, which may be applied not only for NP targeting, but also in hyperthermia therapy, with important effects in the treatment of malignant tumors [37]. When exposed to alternating external magnetic field, Fe_3_O_4_ may promote a local temperature increase from 43 to 46 °C [56]. This phenomenon not only contributes to cancerous cells death, but directly affects the NO release pattern from the polymeric coating, as S-nitrosothiols decomposition is directly affected by light, pH, and temperature [57]. In this sense, it is important to highlight that future studies regarding the NO release modulation from Fe_3_O_4_@Ag/CS-NO NPs and the combination with other therapeutic mechanisms of the nanomaterials synthesized in this work might comprehend important advances in the biomedical field.

### Hemolysis of whole blood

When distributed in blood circulation, NPs may also interact with other components that could lead to the destruction of red blood cells, resulting in serious pathologic conditions such as thrombotic disorders, caused by the hemostatic dysregulation, anemia, or renal failure [58]. Therefore, the NPs interaction with plasma proteins and hemolysis of whole blood comprehends a fundamental preclinical evaluation of the NPs’ biocompatibility [59]. In this sense, the hemolytic toxicity of Fe_3_O_4_ NPs, Fe_3_O_4_@Ag NPs, Fe_3_O_4_@Ag/TCS NPs, and Fe_3_O_4_@Ag/CS-NO NPs and the influence of each layer added to the Fe_3_O_4_ NPs in the % of hemolysis of whole blood was investigated. Rat blood was incubated with each type of NP at 10, 50, 75, 100, and 200 µg mL^−1^, in the range of the cell viability assays.

**Fig. 6:**
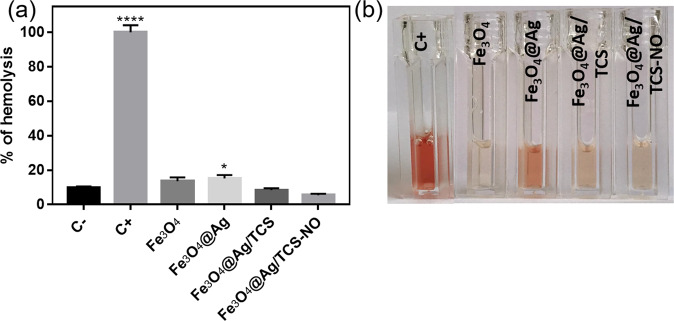
**a** Hemolysis ratio at 50 µg mL^−1^ for negative control (C−), positive control (C+), Fe_3_O_4_@Ag NPs, Fe_3_O_4_@Ag/TCS NPs, and Fe_3_O_4_@Ag/TCS-NO NPs. *P < 0.0332, and ****P < 0.0001, in relation C−; **b** representative image of results obtained for the nanoparticles at the condition shown in the graphic

Figure S4 shows the % of hemolysis for each NP at different concentrations. It was possible to observe that Fe_3_O_4_ NPs did not present concentration-dependent hemolysis, as it was kept constant from the lowest to the highest evaluated concentration. The observed result might be related to the aggregation of uncoated Fe_3_O_4_ NPs, as these NPs tend to present high ratio of aggregation without polymeric or metallic coatings [60]. On the opposite, the increasing concentration of AgNPs, TCS and NO had a direct relation to the hemolysis percentage, similar to the previously reported in literature for pure AgNPs [2]. Furthermore, at low concentrations (10–50 µg mL^−1^), no significant difference (*p* < 0.05) were observed between the NPs, as all presented hemolysis percentages between 5 and 15%. At higher concentrations (75–200 µg mL^−1^), the hemolysis ratio was higher for the NPs with more layers, which is in accordance to cell viability results previously presented. Figure 6 shows the hemolysis ratio (%) for all NPs at 50 µg mL^−1^. It is possible to observe small hemolysis ratio for all NPs, especially for Fe_3_O_4_@Ag/TCS NPs, and Fe_3_O_4_@Ag/CS-NO NPs, suggesting that, at low concentrations, the addition of a polymeric layer, and the NO release corroborate to a higher hemocompatibility of the NPs. Thus, considering a promising range for antitumoral applications and low hemolysis ratio at 50 µg mL^−1^, Fe_3_O_4_@Ag/CS-NO NPs is a promising candidate for localized cancer treatment.

## Conclusion

The results obtained from the combination of Fe_3_O_4_, AgNPs, and NO demonstrated a potential application for a combined treatment with antibacterial and antitumor capacity. The design of a functionalized hybrid NP was strategically developed to achieve a superparamagnetic behavior, due to presence of Fe_3_O_4_ core, allowing to easily guide the NPs upon the application of an external magnetic field, in addition to the functionalization of the magnetic core with AgNPs and NO, both with known antibacterial and antitumoral effects. As expected, the final NP was superparamagnetic and able to delivery therapeutic relevant levels of NO (1.21 ± 0.1 µmol de of NO per mg of the NP). This nanoplatform presented both antibacterial and antitumoral properties, highlighting the bactericidal activity against *E. coli* and an improved cytotoxic and cytostatic effects against MG63 tumoral cell line. Moreover, the hemolytic potential of the Fe_3_O_4_@Ag/CS-NO NPs evidenced an ideal range for future studies, in a safe manner. Therefore, based on the promising results observed for these NPs, our group is further investigating the stability of these NPs under physiological conditions, in addition to the studies of blood protein interactions and inflammatory responses resultant of the impact of these NPs in the biological system.

## Supplementary information

Supplementary Information

## References

[1] Doane TL, Burda C (2012). The unique role of nanoparticles in nanomedicine: Imaging, drug delivery and therapy. Chem Soc Rev.

[2] Chen LQ, Fang L, Ling J, Ding CZ, Kang B, Huang CZ (2015). Nanotoxicity of silver nanoparticles to red blood cells: size dependent adsorption, uptake, and hemolytic activity. Chem Res Toxicol.

[3] Prado-Prone G, Silva-Bermudez P, Almaguer-Flores A, García-Macedo JA, García VI, Rodil SE (2018). Enhanced antibacterial nanocomposite mats by coaxial electrospinning of polycaprolactone fibers loaded with Zn-based nanoparticles. Nanomed Nanotechnol Biol Med.

[4] Mobarki N, Almerabi B, Hattan A (2019). Antibiotic resistance crisis. Int J Med Dev Ctries.

[5] Bonilla-Gameros L, Chevallier P, Sarkissian A, Mantovani D (2020). Silver-based antibacterial strategies for healthcare-associated infections: processes, challenges, and regulations. An integrated review. Nanomed Nanotechnol Biol Med.

[6] Jia X, Zhang Y, Zou Y, Wang Y, Niu D, He Q (2018). Dual intratumoral redox/enzyme-responsive NO-releasing nanomedicine for the specific, high-efficacy, and low-toxic cancer therapy. Adv Mater.

[7] Blanco E, Shen H, Ferrari M (2015). Principles of nanoparticle design for overcoming biological barriers to drug delivery. Nat Biotechnol.

[8] Yuan M, Wang Y, Qin YX (2019). Engineered nanomedicine for neuroregeneration: light emitting diode-mediated superparamagnetic iron oxide-gold core-shell nanoparticles functionalized by nerve growth factor. Nanomed Nanotechnol Biol Med.

[9] Wu K, Su D, Liu J, Saha R, Wang JP (2019). Magnetic nanoparticles in nanomedicine: a review of recent advances. Nanotechnology.

[10] Zhu K, Ju Y, Xu J, Yang Z, Gao S, Hou Y (2018). Magnetic nanomaterials: chemical design, synthesis, and potential applications. Acc Chem Res.

[11] Mahmoudi M, Hofmann H, Rothen-Rutishauser B, Petri-Fink A (2012). Assessing the in vitro and in vivo toxicity of superparamagnetic iron oxide nanoparticles. Chem Rev.

[12] Alcantara D, Lopez S, García-Martin ML, Pozo D (2016). Iron oxide nanoparticles as magnetic relaxation switching (MRSw) sensors: current applications in nanomedicine. Nanomedicine Nanotechnology. Biol Med.

[13] Chen Y, Xianyu Y, Wang Y, Zhang X, Cha R, Sun J (2015). One-step detection of pathogens and viruses: combining magnetic relaxation switching and magnetic separation. ACS Nano.

[14] Martinez-Boubeta C, Simeonidis K, Makridis A, Angelakeris M, Iglesias O, Guardia P (2013). Learning from nature to improve the heat generation of iron-oxide nanoparticles for magnetic hyperthermia applications. Sci Rep..

[15] Laurent S, Saei AA, Behzadi S, Panahifar A, Mahmoudi M (2014). Superparamagnetic iron oxide nanoparticles for delivery of therapeutic agents: opportunities and challenges. Expert Opin Drug Deliv.

[16] Kumar S, Shukla A, Baul PP, Mitra A, Halder D (2018). Biodegradable hybrid nanocomposites of chitosan/gelatin and silver nanoparticles for active food packaging applications. Food Packag Shelf Life.

[17] Zhou Y, Tang RC (2018). Facile and eco-friendly fabrication of AgNPs coated silk for antibacterial and antioxidant textiles using honeysuckle extract. J Photochem Photobio B Biol.

[18] Kraeling MEK, Topping VD, Keltner ZM, Belgrave KR, Bailey KD, Gao X (2018). In vitro percutaneous penetration of silver nanoparticles in pig and human skin. Regul Toxicol Pharm.

[19] Burdușel AC, Gherasim O, Grumezescu AM, Mogoantă L, Ficai A, Andronescu E (2018). Biomedical applications of silver nanoparticles: an up-to-date overview. Nanomaterials.

[20] Anitha A, Deepa N, Chennazhi KP, Nair SV, Tamura H, Jayakumar R (2011). Development of mucoadhesive thiolated chitosan nanoparticles for biomedical applications. Carbohydr Polym.

[21] Riganti C, Miraglia E, Viarisio D, Costamagna C, Pescarmona G, Ghigo D (2005). Nitric oxide reverts the resistance to doxorubicin in human colon cancer cells by inhibiting the drug efflux. Cancer Res.

[22] Chen M, Song F, Liu Y, Tian J, Liu C, Li R (2019). A dual pH-sensitive liposomal system with charge-reversal and NO generation for overcoming multidrug resistance in cancer. Nanoscale R Soc Chem.

[23] Pieretti JC, Rolim WR, Ferreira FF, Lombello CB, Nascimento MHM, Seabra AB (2020). Synthesis, characterization, and cytotoxicity of Fe_3_O_4_@Ag hybrid nanoparticles: promising applications in cancer treatment. J Clust Sci.

[24] Pelegrino MT, de Araújo DR, Seabra AB (2018). S-nitrosoglutathione-containing chitosan nanoparticles dispersed in pluronic F-127 hydrogel: potential uses in topical applications. J Drug Deliv Sci Technol.

[25] Melvin P, Weinstein MD. Methods for Dilution Antimicrobial Susceptibility Tests for Bacteria That Grow Aerobically Clinical and Laboratory Standard Institute, 2019, 11th Edition.

[26] Page B, Page M, Noel C (1993). A new fluorometric assay for cytotoxicity measurements in vitro. Int J Oncol.

[27] Veisi H, Ghorbani F (2017). Iron oxide nanoparticles coated with green tea extract as a novel magnetite reductant and stabilizer sorbent for silver ions: synthetic application of Fe_3_O_4_@green tea/Ag nanoparticles as magnetically separable and reusable nanocatalyst for reduction of 4-nitrophenol. Appl Organomet Chem.

[28] Pachla A, Lendzion-Bieluń Z, Moszyński D, Markowska-Szczupak A, Narkiewicz U, Wróbel RJ (2016). Synthesis and antibacterial properties of Fe_3_O_4_-Ag nanostructures. Pol J Chem Technol.

[29] Grosvenor AP, Kobe BA, Biesinger MC, McIntyre NS (2004). Investigation of multiplet splitting of Fe 2p XPS spectra and bonding in iron compounds. Surf Interface Anal.

[30] Ivashchenko O, Lewandowski M, Peplińska B, Jarek M, Nowaczyk G, Wiesner M (2015). Synthesis and characterization of magnetite/silver/antibiotic nanocomposites for targeted antimicrobial therapy. Mater Sci Eng C.

[31] Fang W, Zheng J, Chen C, Zhang H, Lu Y, Ma L (2014). One-pot synthesis of porous Fe_3_O_4_ shell/silver core nanocomposites used as recyclable magnetic antibacterial agents. J Magn Magn Mater.

[32] Ramesh R, Geerthana M, Prabhu S, Sohila S (2017). Synthesis and characterization of the superparamagnetic Fe_3_O_4_/Ag nanocomposites. J Clust Sci Springe US.

[33] Rolim WR, Pelegrino MT, de Araújo Lima B, Ferraz LS, Costa FN, Bernardes JS (2019). Green tea extract mediated biogenic synthesis of silver nanoparticles: Characterization, cytotoxicity evaluation and antibacterial activity. Appl Surf Sci.

[34] Wang X, Deng A, Cao W, Li Q, Wang L, Zhou J (2018). Synthesis of chitosan/poly (ethylene glycol)-modified magnetic nanoparticles for antibiotic delivery and their enhanced anti-biofilm activity in the presence of magnetic field. J Mater Sci.

[35] Pham XN, Nguyen TP, Pham TN, Tran TTN, Tran TVT (2016). Synthesis and characterization of chitosan-coated magnetite nanoparticles and their application in curcumin drug delivery. Adv Nat Sci Nanosci Nanotechnol.

[36] Iglesias GR, Delgado AV, González-Caballero F, Ramos-Tejada MM (2017). Simultaneous hyperthermia and doxorubicin delivery from polymer-coated magnetite nanoparticles. J Magn Magn Mater.

[37] Pelegrino MT, Pieretti JC, Nakazato G, Gonçalves MC, Moreira JC, Seabra AB (2021). Chitosan chemically modified to deliver nitric oxide with high antibacterial activity. Nitric Oxide Biol Chem.

[38] Costa P, Lobo JMS (2001). Modeling and comparison of dissolution profiles Paulo. Eur J Pharm Sci.

[39] Korsmeyer RW, Peppas NA (1981). Effect of the morphology of hydrophilic polymeric matrices on the diffusion and release of water soluble drugs. J Memb Sci.

[40] Hoseinzadeh E, Alikhani MY, Samarghandi MR, Shirzad-Siboni M (2014). Antimicrobial potential of synthesized zinc oxide nanoparticles against Gram positive and Gram negative bacteria. Desalin Water Treat.

[41] Kora AJ, Rastogi L (2013). Enhancement of antibacterial activity of capped silver nanoparticles in combination with antibiotics, on model Gram-negative and Gram-positive bacteria. Bioinorg Chem Appl.

[42] Pieretti JC, Pelegrino MT, Nascimento MHM, Tortella GR, Rubilar O, Seabra AB (2019). Small molecules for great solutions: can nitric oxide-releasing nanomaterials overcome drug resistance in chemotherapy?. Biochem Pharmacol.

[43] Rolim WR, Pieretti JC, Renó DLS, Lima BA, Nascimento MHM, Ambrosio FN (2019). Antimicrobial activity and cytotoxicity to tumor cells of nitric oxide donor and silver nanoparticles containing PVA/PEG films for topical applications. ACS Appl Mater Interfaces.

[44] Qin L, Gao H (2019). The application of nitric oxide delivery in nanoparticle-based tumor targeting drug delivery and treatment. Asian J Pharm Sci.

[45] Senapati S, Mahanta AK, Kumar S, Maiti P (2018). Controlled drug delivery vehicles for cancer treatment and their performance. Signal Transduct Target Ther.

[46] Osborn AJ, Elledge SJ, Zou L (2002). Checking on the fork: the DNA-replication stress-response pathway. Trends Cell Biol.

[47] Ye X, Franco AA, Santos H, Nelson, David M, Kaufman PD (2003). Defective S phase chromatin assembly causes DNA damage, activation of the S phase checkpoint, ans S phase arrest. Mol Cell.

[48] Eom HJ, Choi J (2010). p38 MAPK activation, DNA damage, cell cycle arrest and apoptosis as mechanisms of toxicity of silver nanoparticles in Jurkat T cells. Environ Sci Technol.

[49] Tanner FC, Meier P, Greutert H, Champion C, Nabel EG, Lüscher TF (2000). Nitric oxide modulates expression of cell cycle regulatory proteins: a cytostatic strategy for inhibition of human vascular smooth muscle cell proliferation. Circulation.

[50] Napoli C, Paolisso G, Casamassimi A, Al-Omran M, Barbieri M, Sommese L (2013). Effects of nitric oxide on cell proliferation. J Am Coll Cardiol.

[51] Park EJ, Yi J, Kim Y, Choi K, Park K (2010). Silver nanoparticles induce cytotoxicity by a Trojan-horse type mechanism. Toxicol Vitr.

[52] Dwivedi S, Saquib Q, Al-Khedhairy AA, Ahmad J, Siddiqui MA, Musarrat J (2015). Rhamnolipids functionalized AgNPs-induced oxidative stress and modulation of toxicity pathway genes in cultured MCF-7 cells. Coll Surf B.

[53] Kumar G, Degheidy H, Casey BJ, Goering PL (2015). Flow cytometry evaluation of in vitro cellular necrosis and apoptosis induced by silver nanoparticles. Food Chem Toxicol.

[54] Çiftçi H, Türk M, Tamer U, Karahan S, Menemen Y (2013). Silver nanoparticles: cytotoxic, apoptotic, and necrotic effects on MCF-7 cells. Turkish J Biol.

[55] Fukumura D, Kashiwagi S, Jain RK (2006). The role of nitric oxide in tumour progression. Nat Rev Cancer.

[56] Oltolina F, Peigneux A, Colangelo D, Clemente N, D’Urso A, Valente G (2020). Biomimetic magnetite nanoparticles as targeted drug nanocarriers and mediators of hyperthermia in an experimental cancer model. Cancers MDPI AG.

[57] Singh RJ, Hogg N, Joseph J, Kalyanaraman B (1996). Mechanism of nitric oxide release from S-nitrosothiols. J Biol Chem.

[58] Laloy J, Robert S, Marbehant C, Mullier F, Mejia J, Piret JP (2012). Validation of the calibrated thrombin generation test (cTGT) as the reference assay to evaluate the procoagulant activity of nanomaterials. Nanotoxicology.

[59] Pallotta A, Parent M, Clarot I, Luo M, Borr V, Dan P (2017). Blood compatibility of multilayered polyelectrolyte films containing immobilized gold nanoparticles. Part Part Syst Charact.

[60] Illés E, Tombácz E (2006). The effect of humic acid adsorption on pH-dependent surface charging and aggregation of magnetite nanoparticles. J Colloid Interface Sci.

