# Supplementary material for: Multifunctional hybrid nanoplatform based on Fe3O4@Ag NPs for nitric oxide delivery: development, characterization, therapeutic efficacy, and hemocompatibility
Source: J Mater Sci Mater Med. 2021 Mar 6;32(3):23. doi: 10.1007/s10856-021-06494-x (PMC7936955; doi:10.1007/s10856-021-06494-x)
Supplement: Supplementary file 1 — Supplementary Information [file 10856_2021_6494_MOESM1_ESM.docx]

**Multifunctional hybrid nanoplatform based on Fe_3_O_4_@Ag NPs for nitric oxide delivery: development, characterization, therapeutic efficacy and hemocompatibility**

Joana Claudio Pieretti,^1^ Marcelly Chue Gonçalves,^2^ Gerson Nakazato,^2^ Ana Carolina dos Santos Galvão,^1^ Ariane Boudier,^3^ Amedea Barozzi Seabra^1 *^

^1^ Center for Natural and Human Sciences (CCNH), Federal University of ABC (UFABC), Santo André, SP, Brazil

^2^ Department of Microbiology, Universidade Estadual de Londrina, Londrina, PR, Brazil

^3^ Université de Lorraine, CITHEFOR, F-54000 Nancy, France

*Corresponding author

Amedea B. Seabra

Center for Natural and Human Sciences (CCNH), Federal University of ABC (UFABC)

Av. dos Estados 5001, CEP 09210-580, Santo André, SP, Brazil

E-mail: [amedea.seabra@ufabc.edu.br](mailto:amedea.seabra@ufabc.edu.br)

**Supplementary data**

**1. Materials and methods**

*1.1 Synthesis of Fe_3_O_4_@Ag NPs*

Fe_3_O_4_@Ag NPs were obtained through the green reduction of silver ions on the surface of Fe_3_O_4_ NPs. Firstly, Fe_3_O_4_ NPs were synthesized via chemical co-precipitation, adapted from Santos et al. 2016 [1]. Acid solutions of Fe^2+^ and Fe­^3+^ were mixed in a molar proportion of 2 :1, respectively. A solution of NH_4_OH (0.7 mol L^-1^) was slowly dripped in the iron salts solution, until the formation of Fe_3_O_4_ NPs. The obtained nanoparticles were washed several times with water and ethanol, freeze dried and stored protected from light. To synthesize Fe_3_O_4_@Ag NPs, Fe_3_O_4_ NPs were resuspended in deionized water in ultrasound bath for 1 h and homogenized with silver nitrate (AgNO_3_) solution (0.65 mol L^-1^) for 1 h, protected from light. A volume of 60 mL of green tea extract was added, the pH of the final suspension was adjusted to 11 using NaOH (0.1 mol L^-1^), and the mixture was kept stirring for 2 h. Finally, the nanomaterial was magnetically separated, washed with water, freeze dried and stored protected from light [2].

*1.2 Synthesis of TCS*

The backbone of CS was thiolated leading to the formation of thiolated chitosan (TCS), according Anitha et al. with modifications [3]. CS was solubilized in acetic acid (1%) in a proportion of 1% wt/v. A volume of 0.75 mL of thioglicolic acid and 2.44 g of *N*- (3-dimethylaminopropyl)-*N*'-ethylcarbodiimide hydrochloride were dissolved in 1 mL of water and added to the CS solution. The final mixture was stirred for 4 h, at room temperature. TCS was precipitated using 5 mL of ketone, filtered, washed several times with water, freeze dried, and stored protected from light, at 4 °C for future use.

*1.2 Synthesis of Fe_3_O_4_@Ag/TCS NPs*

For coating Fe_3_O_4_@Ag NPs with the prepared TCS, 0.1 g of of the powdered nanoparticles were resuspended in water, using ultrasound bath for 1 h. Simultaneously, 0.02 g of TCS was dissolved in acetic acid (1%). The solutions were mixed and homogenized for 2 h, leading to Fe_3_O_4_@Ag/TCS NPs. The nanomaterial was magnetically separated, washed with water, freeze dried and stored protected from light.

*1.3 Formation of Fe_3_O_4_@Ag/CS-NO NPs*

Free thiol groups (SH) on the surface of Fe_3_O_4_@Ag/TCS NPs were nitrosated by dispersing the nanoparticles (1 mg) in water, using ultrasound bath for 1 h. An excess of NaNO_2_ solution was added to the nanoparticles in suspension and kept in ultrasound bath for 20 min, at room temperature, and protected from light. Fe_3_O_4_@Ag/CS-NO NPs were centrifuged and washed with deionized water in order to remove NaNO_2_ in excess. Fig.S1 shows the schematic representation of the formation of Fe_3_O_4_@Ag/CS-NO NPs. The extension of the nitrosation reaction was determined by the quantification of the amount of S-nitrosothiol groups (CS-NO) on the surface of the Fe_3_O_4_@Ag/CS-NO NPs by using the Free Radical Analyzer (WPI TBR4100/1025 amperometer World Precision Instruments Inc., Sarasota FL, USA) and a nitric oxide specific ISO-NOP sensor (2 mm). Aliquots of 0.2 mL of the Fe_3_O_4_@Ag/CS-NO NPs suspension were added to the sampling compartment, which contained 10 mL of aqueous solution of copper chloride (0.1 M). This condition allowed for the detection of free NO released from the S-nitrosothiol contents in nanoparticle. The experiments were performed in triplicate, and the calibration curves were obtained with aqueous solutions of freshly prepared S-nitrosoglutathione (data not shown).

_
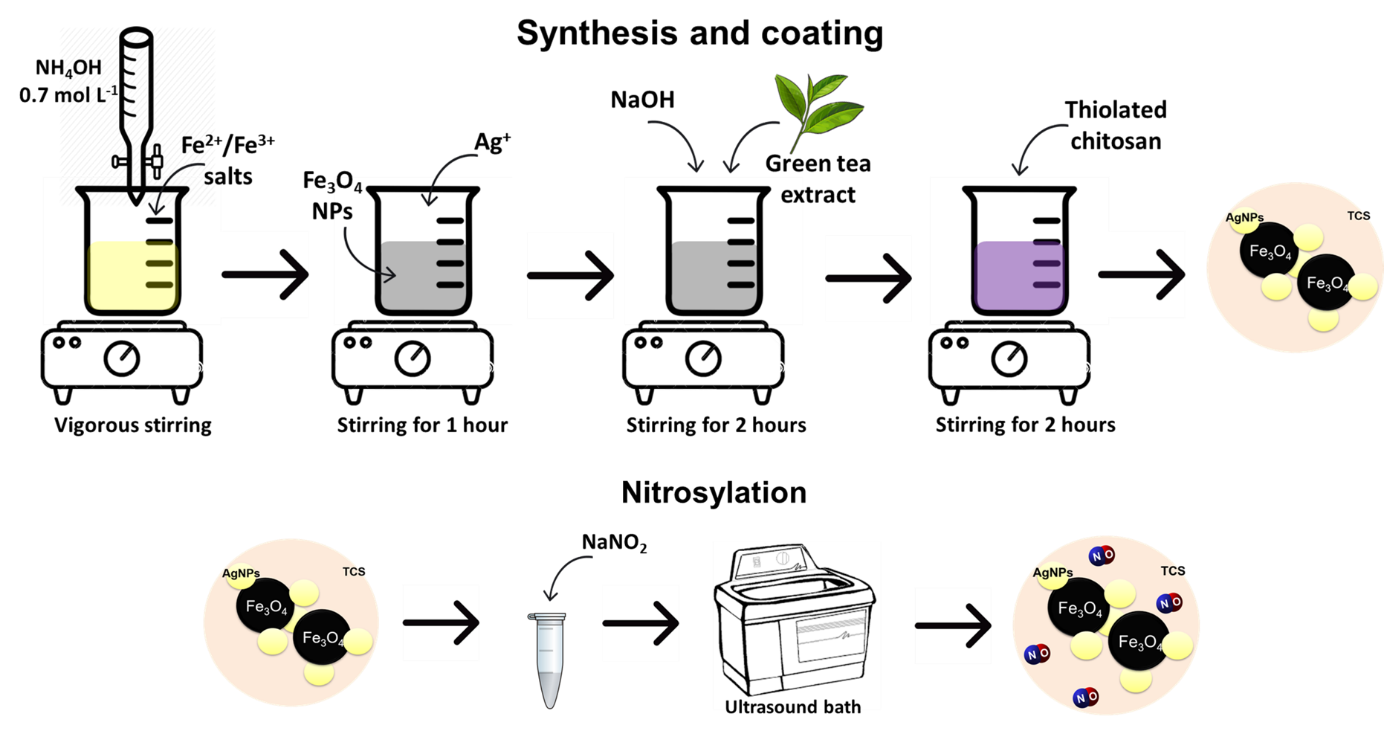
_

**Fig.S1** Schematic representation of synthesis of Fe_3_O_4_@Ag/CS-NO NPs.

*1.4 Characterization of Fe_3_O_4_@Ag/TCS NPs and control NPs*

The structure of Fe_3_O_4_@Ag/TCS NPs was analyzed by XRD, XPS and FTIR. XRD was performed using a STADI-P diffractometer (Stoe®, Darmstadt, Germany). The powdered sample was analyzed in the 2θ range from 5.0° to 64.265°, with step sizes of 0.015º, with MoKα1 (λ = 0.7093 Å) radiation. XPS was used to quantify the elemental composition of the Fe_3_O_4_@Ag/TCS NPs. The measurement was carried using a *K*-alpha spectrometer (*Thermo*, MA, USA) with a monochromated Al K-alpha+ source (E = 1486.6 eV/72 W) with a medium current, 3000 eV energy, 400 µm spotsize and 10 nm of depth. CasaXPS software was used to analyze data.

SEM images of Fe_3_O_4_@Ag/TCS NPs were obtained by a Hitachi-SU3500 (*Hitachi*, Tokyo, Japan), at an accelerating voltage of 10 kV coupled with an EDS at an accelerating voltage of 20 kV. The sample was prepared on a carbon tape attached to an aluminum stub. The EDS technique was used to map the elemental distribution of iron (Fe), oxygen (O), silver (Ag) and sulfur (S). TEM images of Fe_3_O_4_@Ag/TCS NPs were acquired using a JEM 2100 B_6_ transmission electron microscope (JEOL, Akishima, Tokyo), with resolution of 0.25 nm point-to-point and acceleration voltage of 200 kV. Samples were dispersed in water and drop casted onto copper grids for analysis. AFM microscopy was also performed to verify the morphology and size of Fe_3_O_4_@Ag/TCS NPs using an AFM/STM Series 5500 dynamics (*Agilent*, CA, USA). Fe_3_O_4_@Ag/TCS NPs were resuspended in water using ultrasound bath, drop casted onto a silicon wafer, and dried at room temperature. Silicon tips of 4 nm thickness, 30 µm width, 125 µm length, a resonance frequency of 320 kHz, and a force constant of 42 N m^-1^ were employed in the non-contact mode. The images were analyzed using WSxM 5.0 software. The hydrodynamic size (estimated by % of number), surface zeta potential (mV) and polidispersity index (PDI) of Fe_3_O_4_ NPs, Fe_3_O_4_@Ag NPs and Fe_3_O_4_@Ag/TCS NPs were analyzed with a Zetasizer Nano ZS (Malvern Instruments Co, UK). The nanoparticles were resuspended in water and evaluated at 25 °C, using disposable folded capillary zeta cell (10 mm path length) and a fixed angle of 173°.

The magnetic properties of the Fe_3_O_4_ NPs, Fe_3_O_4_@Ag NPs, and Fe_3_O_4_@Ag/TCS NPs were performed by a SQUID magnetometer (Quantum Design, VSM-SQUID), using a fixed temperature of 300 K and oscillating magnetic field from -30 to 30 kOe. The powdered samples were confined in capsules and inserted in a straw holder. The mass composition of the Fe_3_O_4_@Ag and Fe_3_O_4_@Ag/TCS NPs was evaluated by thermogravimetric analysis, employing a TGA Q500 (TA Instruments, DE, USA). Employing a heating ramp of 5 °C per minute from 25 °C to 800 °C under a constant flow of 60 mL min^-1^ N_2_.

*1.5 Antibacterial assays*

Samples were stored at -20 °C, in BHI (Brain Heart Infusion) medium supplemented with 20% glycerol. For reactivation before testing, the samples were plated on MH (Müller Hinton) agar, following incubation at approximately 37 °C for 18 h. Using CLSI 2018 (Clinical and Laboratory Standards Institute) standard, before the tests, bacterial concentration was adjusted in 0.5 Mc Farland scale (≅ 1,5x10^8^ CFU mL^-1^) in saline solution, and then diluted to start the experiments with a concentration of approximately 10^5^ CFU mL^-1^.

MIC was executed in a 96 wells plate, using MH broth, testing nanoparticle concentrations from 7.8 to 500 µg mL^-1^ against 10^5^ CFU mL^-1^ (initial inoculum) of each microorganism. The plates were incubated at 37 °C for 24 h, and the concentration with no broth turbidity was determined as the MIC. Aliquots of 10 µL of each well were plated in MH agar and these plates incubated overnight at 37 °C for observation of MBC. The minimal concentration with no bacterial growth on the MH agar plates was determined as the MBC. For time-kill curves, 100 µL of each nanoparticle diluted in MH broth, at MIC concentration, were placed in microtubes with 100 µL of bacteria 10^5^ CFU mL^-1^ in MH broth. These microtubes were incubated at 37 °C, and the content of the microtubes was diluted in saline and plated on MH agar at different incubation times: time zero, after 2 h and after 5 h of incubation. The plates were incubated for 18 h at 37 °C for the analysis of the antibacterial activity of the nanoparticles by counting colonies that survived the treatments.

**2. Results and Discussion**

*2.1 Preparation and characterization of Fe_3_O_4_@Ag/CS-NO NPs*

Fig.S2 shows the (a) SEM images coupled with EDS and (b) AFM micrographs of Fe_3_O_4_@Ag/TCS NPs. In Fig.S2a, it is possible to observe the presence of iron (Fe), oxygen (O), silver (Ag) and sulfur (S) atoms, confirming the presence of the elements of each layer of Fe_3_O_4_@Ag/TCS NPs. AFM micrograph of Fe_3_O_4_@Ag/TCS NPs indicates the formation of spherical nanoparticles, corroborating with TEM image. Moreover, it is possible to observe an outer layer, referrent to the TCS coating. The coating is detectable due to a lower definition resultant of a weaker interaction between the tip an the organic content, when compared to the tip-metal interaction [4].


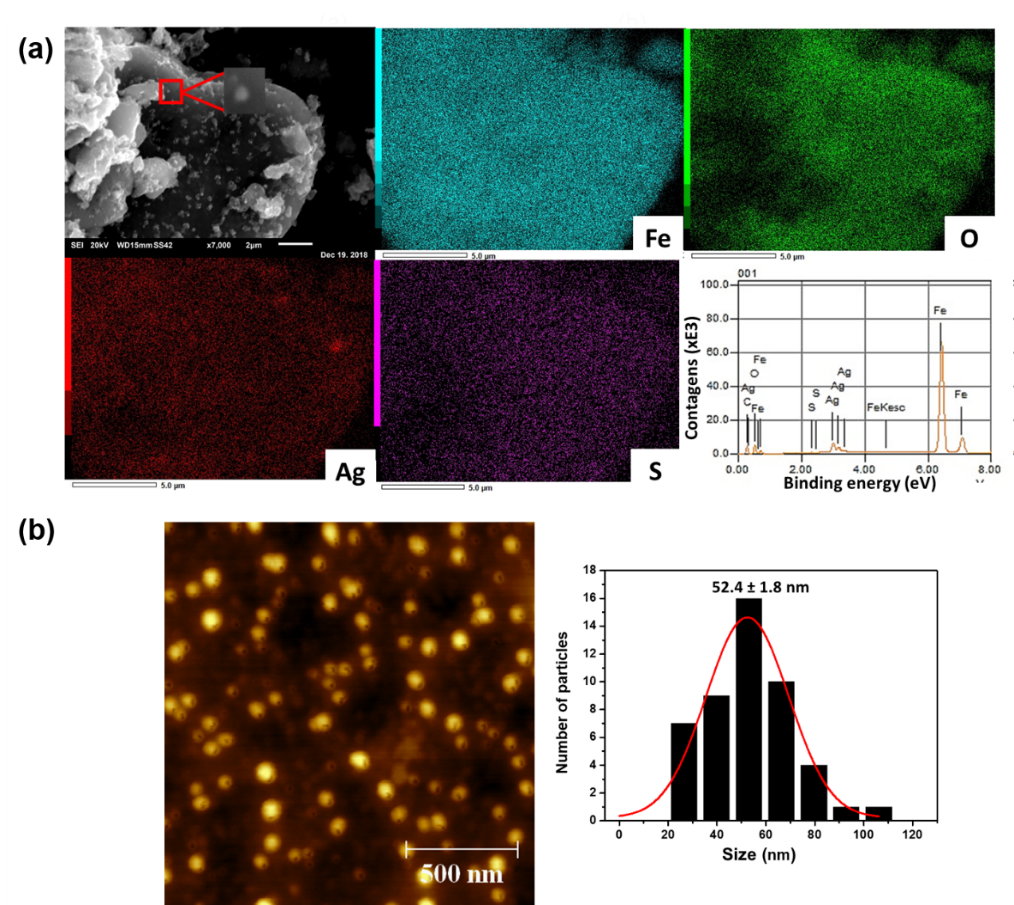


**Fig.S2** (a) SEM images of Fe_3_O_4_@Ag/TCS NPs, elemental mapping showing Fe, O, Ag and S, and the graphic representation; (b) AFM micrograph of Fe_3_O_4_@Ag/TCS NPs and size distribution.

*2.2 In vitro cytotoxicity*

The cytotoxicity of Fe_3_O_4_@Ag NPs, Fe_3_O_4_@Ag/TCS NPs and Fe_3_O_4_@Ag/CS-NO NPs were evaluated against prostate cancer cells (PC3), foreskin fibroblast cells (HFF-1), and epithelial cells (Vero) with 24 h incubation. Fig.S3 evidences that different cytotoxicity are observed when different cell lineages are exposed to the same nanoparticles. Similar pattern has already been observed for different nanoparticles, as toxicity and cellular uptake are dependent on each cell’s tissue source and properties [5,6]. The concentration-dependent cytotoxicity was maintained for all nanoparticles in all three cell lineages shown in Fig.S3 and Fe_3_O_4_@Ag/CS-NO NPs showed the highest toxicity for PC3 and HFF-1 cells. Comparing the cytotoxicity of tumoral PC3 cell line to non-tumoral HFF-1 and Vero cell lines, it is possible to confirm ideal treatment concentration, as previously observed for MG63 and MC3T3, between 5-40 µg mL^-1^, in which the cytotoxicity was higher for tumoral cell lines.


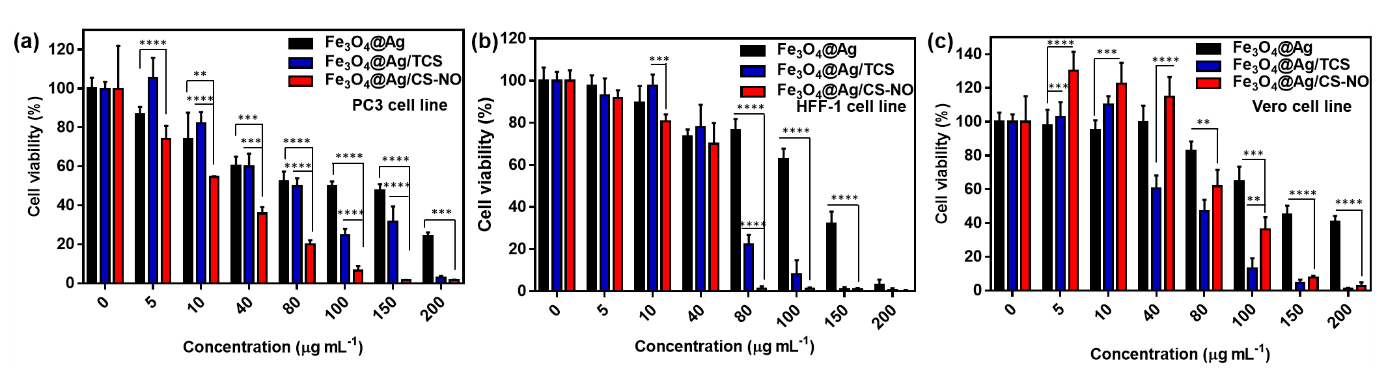


**Fig.S3** Cell viability assays of Fe_3_O_4_@Ag NPs, Fe_3_O_4_@Ag/TCS NPs and Fe_3_O_4_@Ag/CS-NO NPs tested against (a) PC3, (b) HFF-1, and (c) Vero cell lines.

*2.3 Hemolysis of whole blood*


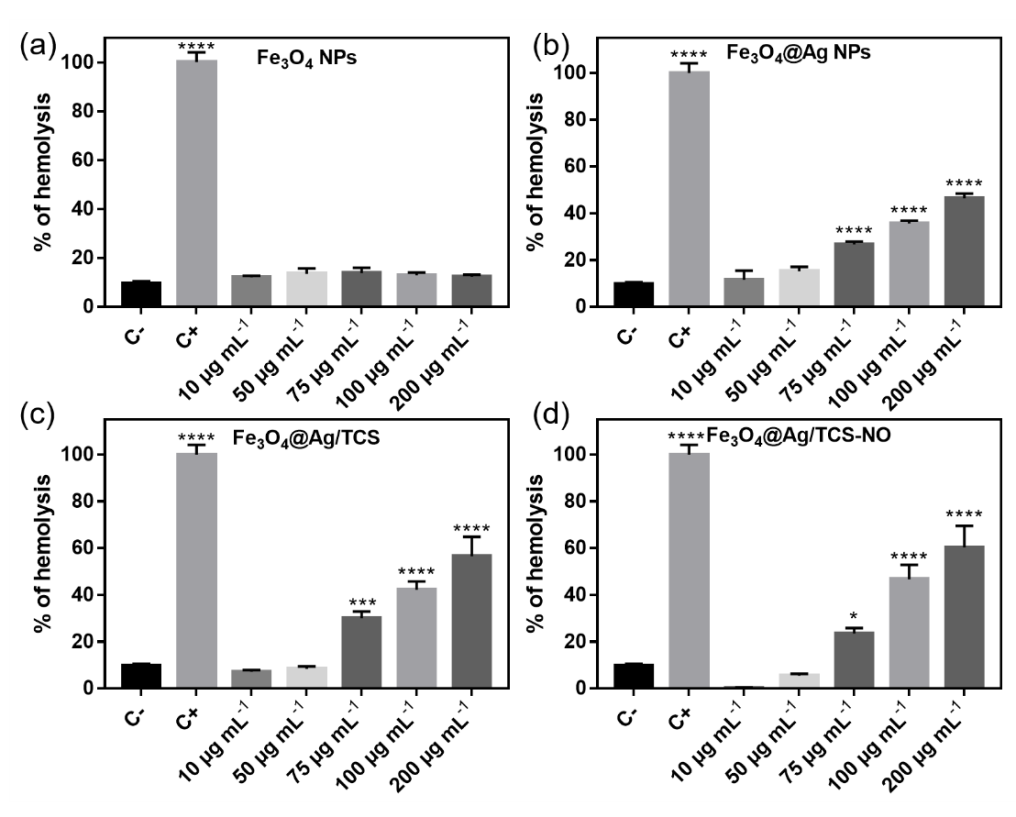


**Fig.S4** Hemolysis ratio in percentage for (A) Fe_3_O_4_ NPs, (B) Fe_3_O_4_@Ag NPs, (C) Fe_3_O_4_@Ag/TCS NPs, and (D) Fe_3_O_4_@Ag/CS-NO NPs. C- represents the whole blood in NaCl 0.9% solution, and C+ represents whole blood in water, considering complete hemolysis of blood in this condition.

**3. References**

1. Santos MC, Seabra AB, Pelegrino MT, Haddad PS. Synthesis, characterization and cytotoxicity of glutathione- and PEG-glutathione-superparamagnetic iron oxide nanoparticles for nitric oxide delivery. Appl Surf Sci. 2016;367:26–35.

2. Pieretti JC, Rolim WR, Ferreira FF, Lombello CB, Nascimento MHM, Seabra AB. Synthesis, Characterization, and Cytotoxicity of Fe_3_O_4_@Ag Hybrid Nanoparticles: Promising Applications in Cancer Treatment. J Clust Sci. 2020;31:535–547.

3. Anitha A, Deepa N, Chennazhi KP, Nair S V., Tamura H, Jayakumar R. Development of mucoadhesive thiolated chitosan nanoparticles for biomedical applications. Carbohydr Polym 2011;83:66–73.

4. Gupta AK, Wells S. Surface-Modified Superparamagnetic Nanoparticles for Drug Delivery: Preparation, Characterization, and Cytotoxicity Studies. IEEE Trans Nanobioscience. 2004;3:66–73.

5. Manshian BB, Soenen SJ, Al-Ali A, Brown A, Hondow N, Wills J, et al. Cell type-dependent changes in cdse/ZnS quantum dot uptake and toxic endpoints. Toxicol Sci. 2015;144:246–58.

6. Chueh PJ, Liang RY, Lee YH, Zeng ZM, Chuang SM. Differential cytotoxic effects of gold nanoparticles in different mammalian cell lines. J Hazard Mater 2014;264:303–12.
